# The emerging role of phosphoinositide clustering in intracellular trafficking and signal transduction

**DOI:** 10.12688/f1000research.7537.1

**Published:** 2016-03-31

**Authors:** Laura Picas, Frederique Gaits-Iacovoni, Bruno Goud

**Affiliations:** 1 Centre de Biochimie Structurale, CNRS UMR 5048, INSERM U1054, Université de Montpellier, Montpellier, France; 2 INSERM, UMR1048, Université Toulouse III, Institut des Maladies Métaboliques et Cardiovasculaires, Toulouse, France; 3 Institut Curie, PSL Research University, CNRS UMR 144, Paris, France

**Keywords:** Phosphoinositides, membrane organization, trafficking, signal transduction.

## Abstract

Phosphoinositides are master regulators of multiple cellular processes: from vesicular trafficking to signaling, cytoskeleton dynamics, and cell growth. They are synthesized by the spatiotemporal regulated activity of phosphoinositide-metabolizing enzymes. The recent observation that some protein modules are able to cluster phosphoinositides suggests that alternative or complementary mechanisms might operate to stabilize the different phosphoinositide pools within cellular compartments. Herein, we discuss the different known and potential molecular players that are prone to engage phosphoinositide clustering and elaborate on how such a mechanism might take part in the regulation of intracellular trafficking and signal transduction.

## Introduction

**Figure 1.**
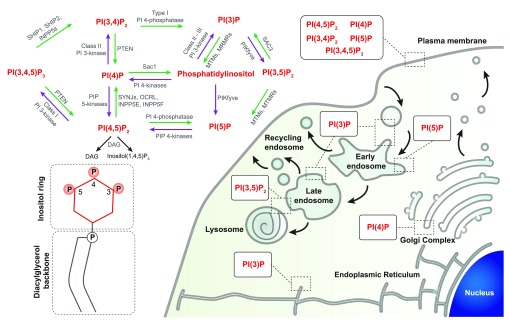
The seven phosphoinositide isoforms identified in eukaryotic cells are phosphorylated derivatives of phosphoinositols, which can be metabolized by different phosphatases and kinases. Representation of the phosphatidylinositol phospholipid structure: the inositol ring can be phosphorylated in three different positions and is linked to a diacylglycerol backbone by a phosphodiester linker. Schematics of the localization of the different PI isoforms on the cellular compartments.

Phosphoinositides (PIs) are essential phospholipids that control, either directly or indirectly, multiple cellular functions including membrane trafficking, signal transduction, cell growth, cytoskeletal dynamics, lipid transport/exchange between organelles, and the regulation of transmembrane proteins ^1, 2^. PIs are the phosphorylated products of phosphatidylinositol. The reversible phosphorylation of the inositol ring at positions 3, 4, and 5 gives rise to the seven PI isoforms identified in eukaryotic cells ( Figure 1). Inter-conversion of the phosphate group(s) is selectively tuned by numerous kinases and phosphatases, precisely regulated in space and time ^3^ ( Figure 1). The active metabolism of PIs is intimately linked to their role as precursors of second messengers during signal transduction ^4^. The accumulation of the different PI species in specific membrane compartments is also directly related to their role in vesicular trafficking including endocytosis and exocytosis, endosome dynamics and trafficking from and towards the Golgi, among many others ^5^ ( Figure 1). Proteins with multiple trafficking functions are targeted to various membrane compartments based on the selective recognition of their PI-binding motifs. The distribution of protein residues folded in a 3D structure provides the PI-binding motifs with a “PI code”, which is based on the stereospecific recognition of the unique phosphate group’s organization around the inositol ring ^6^ ( Figure 1). There are at least 11 different structured motifs with a wide range of affinities and specificities for the different PI species. They include the PH ( pleckstrin homology), the FYVE ( Fab1, YOTB, Vac1, and EEA1), the PX ( Phox homology), the ANTH and ENTH ( AP180 and Epsin N- terminal homology), and the FERM (4.1, ezrin, radixin, moesin) modules.

## PIs and the lateral organization of membranes: the needle in a haystack

Cellular membranes are highly heterogeneous composites built of different types of lipids and proteins. For instance, in eukaryotic cells, more than 1000 different lipid species build up the different membrane compartments ^7^. Lipid molecules freely diffuse in the 2D membrane plane (D ~2.6 × 10 ^-7^ cm ^2^·s ^-1^) ^8^ and interact with protein effectors based on their association ( *K*_on_) and dissociation ( *K*_off_) rates. As a result, lipid-protein interactions are, in general, highly dynamic and thus strongly depend on their respective local concentration.

PIs constitute less than 1% of the steady-state cell lipids ^7^, yet they work as unique docking sites for the multiple PI effectors on membranes, which in turn either compete or cooperate with each other to interact with downstream partners and elicit specific responses. Thus, what are the driving mechanisms that ensure such a thorough spatiotemporal recognition and membrane association of host PI-binding motifs?

An attractive hypothesis is that PIs might be organized as specialized membrane subdomains with distinct organelle localizations ^5^. PI pools within the same compartment are locally synthesized thanks to the spatiotemporal regulation of different PI-metabolizing enzymes ^3, 5^. In addition, small GTPases of the ARF and RAB family also contribute to the generation and regulation of PI turnover on membranes ^9^.

Considering the diffusion coefficient of lipid molecules within the membrane plane, it is likely that complementary mechanisms need to operate in order to spatially preserve the turnover of different PI subdomains. Indeed, several mechanisms have been reported in the literature to play roles as selective and reversible PI sinks by locally sequestering and releasing PIs. This is the case for the myristoylated alanine-rich C-kinase substrate (MARCKS) protein and the growth-associated protein 43 (GAP43) ^10^. The unstructured basic cluster on the effector domain of the MARCKS protein is able to bind up to at least three PI(4,5)P _2_ molecules by means of nonspecific electrostatic interactions at physiologic pH. The Ca ^2+^/calmodulin complex reversibly controls the association of MARCKS with the plasma membrane ^11^. Interestingly, a growing number of studies report the local enrichment of PI subdomains independently of the catalytic activity of PI-metabolizing enzymes. Jahn and co-workers have shown that the SNAP receptor protein syntaxin-1A co-clusters with PI(4,5)P _2_ *via* electrostatic interactions with its juxtamembrane polybasic sequence ^12^. The segregation of PI(4,5)P _2_ microdomains by syntaxin-1A has been proposed to work as a molecular beacon at sites of synaptic vesicle docking during exocytosis ^13^. Similar polybasic clusters to that of the MARCKS protein or syntaxin-1A are found in the cytosolic membrane interface of many plasma membrane proteins ^14, 15^, including the epidermal growth factor receptor (EGFR) and the NMDA receptor as well as the voltage-gated potassium and calcium ion channels ^11^. *In vitro* studies have shown that divalent cations such as Ca ^2+^ are also capable of clustering together PI(4,5)P _2_ molecules, although the exact correlation with the activity of ion channels inside the cell has yet to be established. Following *in vitro* approaches on giant unilamellar vesicles (GUVs), clustering of PI(4,5)P _2_ was initially reported for ezrin ^16^. Later on, using the yeast endocytic F-BAR/BAR domains, Lappalainen and co-authors have shown that the scaffolding effect of these proteins leads to the formation of stable PI(4,5)P _2_ microdomains with reduced lateral diffusion in the membrane plane ^17, 18^. Since then, the list of proteins involved in the formation of PI(4,5)P _2_ clusters has been extended to other endocytic proteins such as Epsin2, AP180, and the N-BAR domain proteins amphiphysin1 and BIN1 ^19^. So far, the formation of PI clusters has been mainly restricted to PI(4,5)P _2_, possibly owing to its multiple regulatory functions at the plasma membrane. In addition, PI(4,5)P _2_ is more abundant than other more elusive PI isoforms and has therefore been the focus of many studies for several years. However, we recently reported that the monophosphate PIs PI4P and PI5P can also be clustered ^19^.

## PI clustering is a diffusion-driven process

PI clustering has initially been proposed to originate from electrostatic interactions and, to a lesser extent, from hydrogen bonding between PI headgroups. PI molecules appear thus sequestered beneath positively charged surfaces, which results in a significant reduction of lateral diffusion in the membrane plane ^17^. The number of PI molecules that interact with basic residues is determined by the negative net charge of the PIs at a given pH. For instance, the charge of the PI(4,5)P _2_ molecules at pH 3 is −1.5e, whereas at pH 7.4, which is close to the pH of the cytosol (7.2), it is −4e ^20^. For a N-BAR homodimer of charge +8e, one could estimate that at cytosolic pH, the stoichiometry of PI-interacting molecules per protein module is 2:1, which gives an estimated 1.5-fold increase of local PI(4,5)P _2_. However, experimental studies have shown that the binding of the N-BAR module on PI-containing membranes induces a local enrichment of at least 10-fold ^19^. How could such a difference in the local PIs’ enrichment be explained?

**Figure 2.**
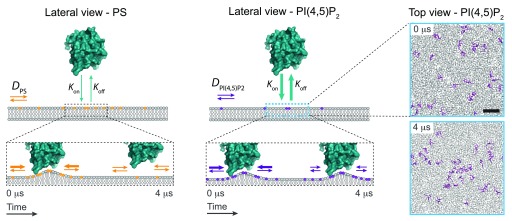
Schematic representation on how phosphoinositide (PI)-binding motifs can engage local demixing of PIs on cellular membranes. As an example, lateral view of the ENTH domain of Epsin (PDB code 1H0A) in cyan upon binding to a membrane that contains monovalent lipids such as phosphatidylserine (PS) (in orange, left panel) or PI(4,5)P _2_ (in magenta, right panel). Cyan arrows represent the *K*_on_/ *K*_off_ rates of the ENTH domain binding on membranes, being faster for PS over PI(4,5)P _2_. As a result, transient demixing of PI(4,5)P _2_ molecules can take place. The diffusion of PS and PI(4,5)P _2_ in the plane of the membrane is depicted by orange and magenta arrows, respectively. Right panel shows a top view of PI(4,5)P _2_ clustering coarse-grain molecular dynamics simulations (as described in 19) on spontaneous membrane biding of an ENTH domain. The panels are snapshots at t = 0 μs and 4 μs of the individual position of PI(4,5)P _2_ molecules (in magenta) along the simulation. Scale bar, 1 nm.

Theoretical studies have shown that the binding of a positively charged protein with a negatively charged membrane induces lipid demixing near to the protein surface ^19, 21^. This phenomenon is the result of the combination of electrostatic interactions and an entropic effect. Upon protein-membrane binding, charged lipids diffuse in the plane of the membrane towards the protein surface to preserve charge neutrality ( Figure 2). In the case of monovalent lipids such as phosphatidylserine (PS), lipid demixing is almost negligible as a result of the fast *K*_on_/ *K*_off_ rates between the protein and the membrane, which prevents charged lipids to locally segregate ^22^ ( Figure 2, left panel). However, for multivalent lipids such as some PI species, the transient interaction with a positively charged protein generates an electrostatic potential well, which results in a reduction of the *K*_on_/ *K*_off_ rates and in protein diffusion. Consequently, transient demixing of PI molecules can take place ^22^ ( Figure 2, right panel). As shown by numerical simulations and consistent with the estimated ~10-fold increase from experimental data, PIs can cluster together up to nine lipid molecules per protein module. The trajectory of PI molecules in the plane of the membrane showed the existence of PI-protein dissociation events, thus pointing out that clustered PI molecules are not sequestered ^19^. Importantly, this behavior is observed at initial physiological relevant concentrations of 1% PI(4,5)P _2_.

PI demixing has been reported in both flat and curved membranes. In the latter case, the segregation of PI molecules is likely to be amplified by membrane curvature since it is reported to significantly reduce protein diffusion ^23^ and lipid dynamics ^17^. This is in agreement with recent molecular simulations that show that clustering of lipids such as PIs and GM3 correlates with membrane curvature ^8^.

## The “PI clustering” toolbox: electrostatic interactions and beyond

Local segregation of PIs into submicron domains has been mostly described for proteins with the intrinsic property to polymerize on membranes, such as the BAR domain family. Proteins of the BAR family can sense and generate membrane curvature, owing to the scaffolding structure that results from the homodimerization of the BAR module. Association of BAR proteins with membranes takes place through electrostatic interactions between positively charged amino acids on the concave/convex face of the dimeric module and acidic phospholipids ^24^. PI clustering has been reported for proteins with F-BAR, BAR, N-BAR, and I-BAR modules ^17– 19^. The tendency of multivalent PIs to engage lipid demixing over the monovalent PS provides BAR proteins with some specificity to generate PI subdomains at the plasma membrane, where PI(4,5)P _2_ and PI(3,4,5)P _3_ are the predominant affected PI isoforms. According to the structural homology within members of the BAR superfamily, it is likely that the formation of PI-enriched microdomains could be a general feature of any protein hosting a BAR module. Combination of the BAR module with PI-binding motifs within the same protein might provide an additional layer of regulation and, possibly, production of monophosphate PI pools in other organelles than the plasma membrane, as observed in the case of BIN1 ^19^. This suggests that the property of PI clustering might be extrapolated to some members of the sorting nexin (SNX) family holding a BAR module and a PX motif ^25^, although this link has yet to be established.

The clustering of PIs is, however, not necessarily associated with the intrinsic ability of proteins to self-assemble. Indeed, the transient segregation of PIs is likely to generate a positive feedback loop. As a result, proteins that selectively interact with PIs can locally accumulate on PI-enriched areas, independently of their ability to polymerize, as observed for the ENTH and ANTH domains ^19^. Therefore, PI clustering seems to be a general property of proteins that directly interact with PIs *via* electrostatic interactions with more or less specificity for a given PI isoform. Accordingly, natively unstructured polybasic protein domains have also been shown to induce local segregation of PIs at the plasma membrane, as observed for MARCKS, GAP43, CAPS23, and syntaxin-1A ^10, 13^. The number of proteins that associate with acidic lipids at the plasma membrane through polybasic sequences is large ^14, 15^. For instance, several small GTPases have been shown to interact with plasma membrane PI(3,4,5)P _3_ and PI(4,5)P _2_ by means of polybasic clusters ^26^.

PI clustering might be solely limited to ionic protein-lipid interactions, although it is tempting to speculate that alternative or complementary mechanisms might take on the stabilization of PI pools. For instance, recent studies have shown that the pinning of the cytoskeleton on membranes preserves liquid-ordered and liquid-disordered (Lo-Ld) phase coexistence at physiological temperatures (37°C) ^27, 28^. The polymerization of actin cytoskeleton was also shown to promote segregation of lipid phases in *in vitro* models ^29^. These observations are in agreement with the “picket fence” model, which predicts that the cytoskeletal network might act as a diffusion barrier for lipids and proteins ^30^. The exact partition of PI(4,5)P _2_ into Lo-Ld domains is not yet clear, but the depletion of cholesterol with methyl-β-cyclodextrin was shown to reduce PI(4,5)P _2_ levels at the plasma membrane ^31^. The partition of PI(4,5)P _2_ to cholesterol-dependent domains was also reported using the targeting of a 5-phosphatase ^32^. In addition, the sequestration of syntaxin-1A microdomains at sites of synaptic vesicle exocytosis in the plasma membrane was shown to require the formation of cholesterol and PI(4,5)P _2_-mediated clusters, which are both distinct from lipid “rafts” ^12, 33^. An interesting observation is that Ld domains were found to align along actin fibers independently of the lipid phase to which actin was pinned ^28^. This might be explained by local changes in membrane curvature induced by the actin network. Indeed, Ld domains appear to favor lipid sorting and membrane deformation ^34^. Recently, numerical simulations have shown that clustering of lipids such as PI(4,5)P _2_ correlates with membrane curvature ^8^. The exact contribution of membrane curvature itself in PI clustering is not yet established, but lipid packing defects associated with membrane curvature might favor a better exposure of PI(4,5)P _2_ headgroups ^19, 35^. Here, one will have to take into account in future experiments the nature of the fatty acids present on PI molecules, which might also impact on the rigidity and shape of the lipid bilayers to which they belong.

## PI clustering: a novel regulator of intracellular trafficking and signaling?

Importantly, after PI clustering, protein-PI dissociation can still take place independently of the initial concentration of PIs ^19^. This suggests that PI clusters are more dynamic than initially anticipated and that a given PI cluster could sequentially interact with different effectors. Thus, PI clustering induced by an upstream protein could favor the recruitment of a downstream PI-binding partner, providing a mechanism to coordinate trafficking or signaling events.

**Figure 3.**
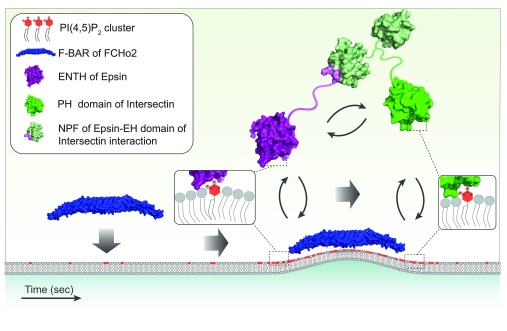
Schematics of the potential role of phosphoinositide (PI) clustering to coordinate cell trafficking events: example of the biogenesis of a clathrin-coated vesicle. The F-BAR domain (Protein Data Bank [PDB] code 2V0O) of FCHo2 binds to the plasma membrane, driving PI(4,5)P _2_ segregation into clusters. The local PI(4,5)P _2_ enrichment drives the binding of Epsin through the interaction of its ENTH domain (PDB code 1H0A) with PI(4,5)P _2_. The Asn-Pro-Phe (NPF) domain of Epsin can interact with the EH domain (PDB code 3FIA) of Intersectin, which in addition hosts a PH domain (PDB code 1MAI) that binds to PI(4,5)P _2_. The dynamics of the system is likely influenced by the affinity constant of the PI(4,5)P _2_-binding motifs, which will determine the K _on_/K _off_ of PI(4,5)P _2_-mediated membrane binding, and by the affinity constant between the different protein domains.

One process that PI clustering could regulate is clathrin-mediated endocytosis (CME). Indeed, the F-BAR, ANTH, ENTH, and N-BAR domains are present in central molecular players involved in CME ^36^. All of these protein modules have been shown to engage local segregation of PI(4,5)P _2_^17, 19^, which is the key PI isoform in CME. Therefore, PI clustering could participate in the spatiotemporal regulation of CME based on the affinity constant of the different protein intermediates and their interaction with PI(4,5)P _2_. A hypothetical example of how PI clustering might operate in CME is shown in Figure 3, although the number of PI(4,5)P _2_ effectors implicated in CME is much larger (see Table 1). The polymerization of the N-BAR module along the bud neck is likely to establish a diffusion barrier ^37^, highly enriched in PIs, which would thereby be shielded from the activity of kinases and phosphatases. These features might be relevant at different stages of clathrin-coated vesicle biogenesis. Indeed, the metabolic evolution of PIs during CME has been shown to be important for the maturation of clathrin-coated vesicles ^38^. In addition, the segregation of lipid phases has been reported to generate sufficient line tension to induce membrane scission ^39^. It is therefore possible that the PI demixing induced by BAR proteins plays an additional role in line tension-mediated fission at the last stage of CME, as suggested by theoretical studies ^40^.

**Table 1.** PI(4,5)P _2_ effectors implicated in CME. The table shows an overview of all the possible options that exist in the PI(4,5)P _2_-mediated protein recruitment during the different stages of CME. Notice that although the interaction with PI(4,5)P _2_ is mostly electrostatically driven, some effectors hold structured motifs with specific affinities/selectivity for PI(4,5)P _2_. In addition, effectors can act as either monomers or larger assemblies, although PI(4,5)P _2_ clustering can engage the local accumulation of proteins that typically do not self-assemble as a result of positive feedback ^19^.

Mammalia n protein	Function	PI(4,5)P _2_ interaction	Self- assembly	References
FCHo 1/2	Membrane curvature (F-BAR)	Charge dependent	Yes	17, 41, 42
AP2	Adaptor complex	α subunit C-μ2 subunit	No	43, 44
Intersectin	Scaffolding protein	PH domain	Yes	45– 47
AP180, CALM	Adaptor of AP2 and clathrin	ANTH domain	No	48, 49
HIP1-HIP1R	Links actin to clathrin	ANTH domain	No	49, 50
Epsin	Membrane bending	ENTH domain	No	49, 51
Amphiphysin	Membrane curvature (N-BAR)	Charge dependent	Yes	19, 24, 52
Endophilin	Membrane curvature (N-BAR)	Charge dependent	Yes	24, 53
Syndapin	Membrane curvature (F-BAR)	Charge dependent	Yes	17, 54, 55
SNX 9/18	Membrane curvature (BAR)	PX domain	Yes	56, 57
Dynamin	Scission	PH domain	Yes	58– 60
OCRL	PI 5-phosphatase	PH domain	No	61
Numb	Cargo adaptor (Notch)	PTB/PI domain	No	62, 63
Dab2	Cargo adaptor (LDLR)	DH domain	No	64– 66
ARH	Cargo adaptor (LDLR)	PTB/PI domain	No	65, 66

It is tempting to propose that the coordinated action of PIs and scaffolding protein complexes, in particular BAR proteins, is a general feature of the biogenesis of transport vesicles ^67^. For instance, the N-BAR protein Arfaptin 1 has been shown to participate in the biogenesis of secretory storage granules through the interaction with PI4P at the *trans*-Golgi network ^68^. The ArfGAP ASAP1 also carries a BAR module along with a PI-binding motif and has been shown to provide a platform to regulate Arf4 and Rab8/Rab11-mediated targeting of rhodopsin transport carriers to cilium ^69^. Finally, some members of the SNX family also hold a BAR module in addition to the characteristic PX domain, which typically binds to PI3P ^6^. The SNX-BAR proteins are implicated in tubule-based endosomal sorting ^70^. This includes the two retromer subunits SNX1 and SNX2, SNX5, and SNX6 or SNX4 among others ^71, 72^. One may speculate that the formation of PI clustering together with the binding affinity for different PI effectors might be linked to the ability of SNX-BAR proteins to define tubular endosomal subdomains.

PI clustering could also play an important role in the coordination of signaling events. Interestingly, the juxtamembrane segment of the EGFR, which is implicated in the activation of the receptor, is also composed of a cluster of basic residues that interact with PI(4,5)P _2_^73, 74^. Indeed, natively unstructured polybasic protein domains have been shown to engage PI(4,5)P _2_ clustering ^11^. The interaction of the EGFR with PI(4,5)P _2_ is required for the activation and downstream signaling of the receptor at the plasma membrane and seems also to regulate its fate in the endosomal compartments. The first observation that PI4P 5-kinase activity generating PI(4,5)P _2_ pools was associated with the EGFR and required for appropriate activation and downstream signaling originates from the early 90s ^75^. Later studies demonstrated that PI(4,5)P _2_ clustering induced by the binding and antiparallel dimerization of the juxtamembrane segments of two associated EGFRs can lead to the activation of the receptor even in the absence of ligand ^76^. This property was suggested to be important at a high density of EGFR monomers (>800/µm ^2^), as is often observed in aberrant activation of the receptor in cancers ^73, 77^. In this condition, formation of EGFR nanoclusters takes place as a result of the electrostatic interaction between PI(4,5)P _2_ molecules at the plasma membrane and the juxtamembrane region of the receptor ^78^.

Recent evidence demonstrates that PI(4,5)P _2_ generated on endosomes is required for the appropriate sorting of active EGFR towards multivesicular bodies and further termination of the signal. This process relies on the recruitment of the endosomal type Iγ PIP kinase, PIPKIγi5, that gets targeted to early endosomes by association with SNX5, an effector of PI(4,5)P _2_. The kinase will then increase local pools of PI(4,5)P _2_, also required for association of SNX5 with Hrs proteins that will then interact with ubiquitinated EGFR and ensure its proper sorting ^79^.

It is noteworthy that most of the tyrosine kinase receptors of the EGFR family harbor a polybasic juxtamembrane domain that could play the same role in terms of ligand free activation or sorting and signal transduction (e.g. insulin-like growth factor 1 receptor [IGF1R], vascular endothelial growth factor receptor [VEGFR], platelet-derived growth factor receptor [PDGFR], and fibroblast growth factor receptor 1 [FGFR1], among others) ^76^. Although PI clustering being a general feature of membrane-associated polybasic domains provides an attractive hypothesis to activate receptors and trigger signaling, work is still needed to define whether it is a broad mechanism or applies only to some specific proteins.

## Conclusions

The spatiotemporal remodeling of PI pools within distinct organelles is an intrinsic feature that makes possible the orchestration of PI-mediated cellular functions. Indeed, PIs are constantly subjected to the activity of PI-metabolizing enzymes and must be in addition accessible to effectors. Because the lateral diffusion of lipid molecules within the membrane plane is extremely fast, PI clustering comes up as a realistic mechanism to locally preserve newly metabolized PI pools on cellular membranes. Indeed, Balla and co-workers already anticipated that PI4P replenishment from the Golgi was not essential to preserve the plasma membrane pool, although it does contribute to its formation ^80^. Irvine and co-authors also showed that the maintenance of the steady-state pool of PI(4,5)P _2_ at the plasma membrane does not require localization of its synthetic precursor PI4P on the same cellular compartment ^81^. It is tempting to speculate that PI clusters might work as potential platforms to coordinate PI-mediated protein interactions or as molecular beacons, as previously proposed ^13^. Nevertheless, the myriad of protein modules capable of engaging PI clustering is becoming broad. Based on structural homologies, one might predict that the list will progressively increase. An interesting feature to point out is that PI clustering seems to be a general mechanism for either multivalent or monophosphate PIs ^19^. The precise regulatory role of PI clustering in trafficking and signal transduction has still to be established, but it certainly opens up exciting perspectives in the field. For instance, PI clustering might orchestrate the different steps in carrier biogenesis. Also, the ability of cellular receptors to engage PI clustering might determine their sorting to the appropriate compartment. The physiological implication of PI clustering in living organisms has yet to be established. Recent studies have already shown that the oligomerization of Sec14-nodulin proteins controls the localization of PI(4,5)P _2_ and signaling landscape in polarized membrane morphogenesis in *Arabidopsis thaliana* root hairs ^82, 83^. Despite the role of PIs in many cellular processes, certain PI isoforms and functions have often been elusive due to the lack of detection or labeling strategies, which is typically limited to the use of PI-binding motifs with all of the associated side effects. The development of novel experimental strategies capable of detecting the intrinsic dynamics of PIs or of exploiting the recently developed sub-100nm life cell imaging techniques ^84^ will be key to unraveling the regulatory role of PI clustering.
